# Towards Automatic Collaboration Analytics for Group Speech Data Using Learning Analytics

**DOI:** 10.3390/s21093156

**Published:** 2021-05-02

**Authors:** Sambit Praharaj, Maren Scheffel, Marcel Schmitz, Marcus Specht, Hendrik Drachsler

**Affiliations:** 1 Educational Science Faculty, Open University of the Netherlands, 6419 AT Heerlen, The Netherlands; marcel.schmitz@zuyd.nl (M.S.); drachsler@dipf.de (H.D.); 2 Institute of Education Science, Ruhr-Universität Bochum, 44801 Bochum, Germany; 3 Information Communication Technology Faculty, Zuyd University of Applied Sciences, 6419 DJ Heerlen, The Netherlands; 4 Computer Science Faculty, Delft University of Technology, 2628 CD Delft, The Netherlands; m.m.specht@tudelft.nl; 5 Information Center for Education, DIPF Leibniz Institute for Research and Information in Education, 60323 Frankfurt am Main, Germany; 6 Computer Science Faculty, Goethe University, 60323 Frankfurt am Main, Germany

**Keywords:** collaboration, collaboration analytics, co-located collaboration analytics, group speech analytics, multimodal learning analytics

## Abstract

Collaboration is an important 21st Century skill. Co-located (or face-to-face) collaboration (CC) analytics gained momentum with the advent of sensor technology. Most of these works have used the audio modality to detect the quality of CC. The CC quality can be detected from simple indicators of collaboration such as total speaking time or complex indicators like synchrony in the rise and fall of the average pitch. Most studies in the past focused on “how group members talk” (i.e., spectral, temporal features of audio like pitch) and not “what they talk”. The “what” of the conversations is more overt contrary to the “how” of the conversations. Very few studies studied “what” group members talk about, and these studies were lab based showing a representative overview of specific words as topic clusters instead of analysing the richness of the content of the conversations by understanding the linkage between these words. To overcome this, we made a starting step in this technical paper based on field trials to prototype a tool to move towards automatic collaboration analytics. We designed a technical setup to collect, process and visualize audio data automatically. The data collection took place while a board game was played among the university staff with pre-assigned roles to create awareness of the connection between learning analytics and learning design. We not only did a word-level analysis of the conversations, but also analysed the richness of these conversations by visualizing the strength of the linkage between these words and phrases interactively. In this visualization, we used a network graph to visualize turn taking exchange between different roles along with the word-level and phrase-level analysis. We also used centrality measures to understand the network graph further based on how much words have hold over the network of words and how influential are certain words. Finally, we found that this approach had certain limitations in terms of automation in speaker diarization (i.e., who spoke when) and text data pre-processing. Therefore, we concluded that even though the technical setup was partially automated, it is a way forward to understand the richness of the conversations between different roles and makes a significant step towards automatic collaboration analytics.

## 1. Introduction

Collaboration is an important 21st Century skill [1]. Basically, collaboration occurs when two or more persons work towards a common goal [2]. The majority of the works in the field of learning analytics to generate collaboration insights have focused on the analysis of distributed (or online) collaboration [3]. However, with the ubiquity of the use of sensors [4,5], multimodal learning analytics [6,7,8] has picked up pace, thus shifting the focus to the analysis of co-located collaboration (CC) (or face-to-face collaboration) with the help of sensor technology [5,9,10,11]. Moreover, sensor technology is scalable [12] and has become affordable and reliable in the past decade [13]. CC takes place in physical spaces where all group members share each other’s social and epistemic space [14]. “The requirement of successful collaboration is *complex, multimodal, subtle,* and learned over a lifetime. It involves *discourse, gesture, gaze, cognition, social skills, tacit practices,* etc.” ([15] pp. 1–2, emphasis added). Therefore, the quality of collaboration can be detected from one or more of the different modalities like audio, video and data logs. Audio is a commonly occurring modality during collaboration [16,17,18,19].

The quality of co-located collaboration from group speech data alone has been detected in the past using indicators of collaboration derived from audio. The indicators of collaboration can be as simple as total speaking time of a group member [17] or as complex as synchrony in the rise and fall of the pitch [20]. For example, Bachour et al. [17] mirrored the total speaking time of each group member in the form of the number of glowing coloured LED lights for each group member on a tabletop display. Then, they found that group members who spoke more reduced their speaking time and group members who spoke less improved their total speaking time. In the other example, two members in a group were speaking at different amplitudes, but exhibiting the same pattern of their speech (e.g., the rise and fall of the average pitch of both members were similar to each other), then they showed a high level of synchrony [20], which resulted in a better rapport and better quality of collaboration. Therefore, the collaboration indicators are context dependent. This is due to the differing goals and fundamental characteristics or parameters (such as group behaviour, interaction, composition) of the group in each collaboration context. The parameters of collaboration are primary aspects such as *team composition* (e.g., experts, initiators or roles of being initiators), the *behaviour of team members* (e.g., dominance, rapport, conflict), the types of interaction (e.g., active or passive) and *behaviour during collaboration* (e.g., knowledge co-construction, reflection, coherence, misconception, uncertainty).

Most studies on CC in the past focused on automated analysis using temporal (time domain features like the energy of the signal, amplitude), spectral indicators of speech (frequency-based features like pitch, rhythm) [16,20] and other *non-verbal indicators* like total speaking time [17,18], frequency of turn taking [21] or using machine learning classifiers to analyse these features of speech [16,22]. Therefore, most of these studies focused on the analysis of the non-verbal indicators of audio instead of looking at the verbal audio indicators such as the content of the conversation, actual keywords used, dialogues and the main themes of conversation. These non-verbal audio indicators do not convey true meaning because most used black-box machine learning methods and some studies reported the indicators (e.g., silence is an indicator for collaboration quality [22]) without informing about the valence, i.e., how good or bad these indicators are. Moreover, the non-verbal audio indicators are less overt as compared to verbal audio indicators. For example, higher or lower total speaking time may be a good or bad indicator of collaboration quality, while “yes” or “no” will most of the time convey the same semantic meaning in any conversation. Few other studies have focused on the non-automated (or semi-automated) coding and analysis of the content of speech, which is laborious [16,20].

Apart from the majority of studies focusing on the analysis of *non-verbal audio indicators*, very few studies used the *verbal audio indicators* or the content of the audio for the analysis of CC quality. For example, for “talk traces” [23] and “meeter” [24], verbal audio indicators of collaboration were used for the analysis. In “talk traces”, Chandrasegaran et al. [23] did topic modelling during the meeting and then showed the topic clusters as a visualization feedback by comparing with the meeting agenda, which was fixed before the meeting. Moreover, topic modelling shows a surface-level analysis based on a collection of representative keywords, which is not rich enough to understand the group conversations in depth. It does not show the proper linkage between these words and the rest of the conversation, which can lead to the loss of the holistic meaning of the conversations and a possible under-representation of certain topics. The other “meeter” study [24] classified the dialogues of the group members based on a lab study to measure information sharing and shared understanding while generating ideas. The collaborative task was based on three open-ended fixed topics where group members needed to brainstorm and share their ideas in a short session of 10 min. Their performance (or quality of collaboration) was measured based on the number of ideas they wrote down on the cards, which was quality controlled before counting the total ideas to weed out bad ideas. They did not find significant effects of information sharing and shared understanding on the quality of collaboration. Therefore, these studies on verbal audio indicators of collaboration were too abstract in either choosing representative keyword clusters as topics or classifying dialogues into a few selected categories that do not affect the collaboration quality. They did not show the linkage of the conversation between different group members. Furthermore, these studies were performed in controlled settings. Therefore, to overcome these limitations, we conducted a field trial to build a technical setup and then prototyped it in real-world settings to advance towards automatic collaboration analytics from group speech data. To this end, we have the following overarching research question:

RQ: To what extent can co-located collaboration analytics from group speech data be done automatically?

To answer this primary research question, we sub-divided it into two sub-research questions:

RQ1: What co-located collaboration indicators have been detected from group speech data in past studies?
RQ2: What collaboration analytics can be employed to analyse group speech data from co-located collaboration automatically?

To answer *RQ1*, we look at the already available literature in Section 2. To answer *RQ2*, we designed a technical setup and report about the materials and methods used in Section 3. Our objective of building this technical setup was to analyse the “what” of the conversation in an automatic manner. We collected, processed and visualized audio data automatically. The data collection took place while a board game was played among the university staff with pre-assigned roles to create awareness of the connection between learning analytics and learning design. This game was also helpful to collect indicators for measuring student and teacher behaviour. We not only did a word-level analysis of the conversations, but also analysed the richness of these conversations by visualizing the strength of the linkage between these words and phrases interactively. Our main goal was to use this technical setup to do role-based profiling based on the exchange of conversation turns taking into account the content of the conversation. To analyse the content of the conversation, we generate meaningful visualizations and interpretations in Section 4. In this visualization, we used a network graph to visualize turn taking exchange between different roles (such as teacher, student and study coach) along with the word- and phrase-level analysis. We also used centrality measures to understand the network graph further based on how much words have hold over the network of words and how influential are certain words. Then, we discuss our findings in Section 5 and the challenges and limitations in Section 6. We found certain limitations of our technical setup in terms of automation in speaker diarization (i.e., who spoke when) and data pre-processing. Finally, we conclude with a highlight of the implications of this work and future work in Section 7. Therefore, the main reason for building this technical setup was to make a starting step towards automatic collaboration analytics, which can assist different stakeholders in a university to understand the group conversations in depth, do role-based profiling and analyse how each group member contributes to the discussion.

## 2. Indicators of Co-Located Collaboration from Audio

Audio is a commonly occurring modality during collaboration [16,17,18,19]. Indicators of collaboration derived from audio are: prosody of sound such as pitch, spectral property, tone and intensity [16]; non-verbal features like total speaking time of group members [17,18], interruptions [25] and overlap or no overlap duration [16]; speaking time of a group member combined with the attention of other group members measured by their eye gaze [19]; linguistic features such as pronouns, sentence length and prepositions [26,27]; verbal features like the keywords used, topics covered [23] and dialogues [24]. It has been found that a combination of both group speech-based and individual speaker-based indicators is a good predictor of the collaboration quality [16]. As seen from the examples in different past studies, these indicators of collaboration are dependent on the context. This is due to the differing goals and fundamental characteristics or parameters (such as group behaviour, interaction, composition) of the group in each collaboration context. The parameters of collaboration are primary aspects such as *team composition* (e.g., experts, initiators or roles of being initiators), the *behaviour of team members* (e.g., dominance, rapport, conflict), the types of interaction (e.g., active or passive) and *behaviour during collaboration* (e.g., knowledge co-construction, reflection, coherence, misconception, uncertainty).

To elaborate further, Terken and Strum [19] designed a mechanism to give real-time feedback to participants in group meetings by analysing their *speaking time* and *eye gaze* behaviour. Feedback was given in the form of different coloured circles representing attention to and from speakers and listeners measured by eye gaze and the total speaking time of that member. This feedback was projected on top of the table in front of where each participant was sitting using a top-down projector. They performed both quantitative and qualitative evaluation of the effect of the feedback: the feedback was accepted as a positive measure by most group members; the use of feedback had a positive impact on the behaviour of group members as they had a balanced participation. There was a balanced participation in terms of the speaking time of each group member. It was found that the eye gaze measured to track the total attention of the listener and speaker was not a good predictor of the quality of collaboration. As per the authors, this was because of the difficulty in intuitively controlling gaze behaviour as compared to controlling the speaking behaviour even though both can be consciously controlled.

Some other works also used total speaking time as an indicator of collaboration [17,18]. The participants were having a group conversation around a smart table. The total speaking time of each member was reflected back to them by a coloured LED light display [17] and concentric circle visualization [18] on the table. They found that this helped to regulate the equality of participation during a group conversation. The group members who spoke most of the time (or were dominant) started to speak less than usual, and the members who spoke less started speaking more, thereby promoting equality of participation among the group members. Therefore, the group that had better equality of speaking time had better quality of collaboration as measured by a post-test.

To analyse other audio indicators in depth, Bassiou et al. [16] used non-verbal features as collaboration indicators. They used a combination of manual annotation and a support vector machine to predict the collaboration quality of the group. The types of collaboration quality marked by expert annotators were: good (when all three members in the group were working together and contributing to the discussion), cold (when only two members were working together), follow (when one member was taking the lead without integrating the whole group) and not (when everyone was working independently). This coding was based on two types of engagement: simple (i.e., talking and paying attention) and intellectual (i.e., actively engaged in the conversation). It was found that a combination of the *group speech activity* indicators (i.e., solo duration, overlap duration of two persons, overlap duration of all three persons, the ratio of the duration of the speaking time of the least and most talkative person in the group, the ratio of the duration of the speaking time of the second most talkative student to the most talkative student in the group) and *individual speaker-based* indicators (i.e., spectral, temporal, prosodic and tonal) were good predictors of collaboration quality as marked by the annotators. Moreover, the group-level indicators alone were good predictors of collaboration quality. According to the authors, this was because the individual speaker-based indicators were agnostic to the group information, contrary to the group speech activity indicators. All these indicators were fed to a machine learning classifier to get the measurements, so in the end, it was a black-box approach. They did not employ any fine-grained analysis, which could help to uncover the degree of contribution of different indicators to the prediction of good or bad collaboration quality.

Similarly, *speaker-based* indicators like the intensity, pitch and jitter were used to detect collaboration quality among working pairs [20]. When two members in a group are speaking at different amplitudes, but exhibiting the same pattern of their speech (e.g., the rise and fall of the average pitch of both members are similar to each other), then they are showing a high level of synchrony [20]. Lubold and Pon-Barry [20] found a positive correlation between synchrony and rapport (generated by comparing perceptual rapport from annotators and self-reported rapport) during collaborative interactions. A good rapport between group members can enhance the collaboration [28]. The prediction gave a high-level overview of non-verbal features like pitch, but missed the fine-grained semantic meaning of different non-verbal features such as turn taking, emotional tone while speaking, cross-talk and number of interruptions. These fine-grained vocal characteristics such as turn taking and overlap of speech are distinctive of collaboration quality; more frequent speaker changes (i.e., *turn taking*) with overlap of speech [21] indicates a good quality of collaboration. Previous research also indicated that overlap in speech is associated with positive group performance [29,30].

Additionally, other works focused on expertise detection and productive problem solving [22,25,31], estimation of success [32], collaboration detection [33] and differentiating student learning strategies [34] during collaboration. Zhou et al. [25] tracked the speech of students working in groups solving math problems. They found that *overlapped speech* was an indicator of constructive problem-solving progress, expertise and collaboration. They used both the *number of overlaps* in speech and the *duration of overlap* in speech when tracking the interruptions during speaking. Luz and Saturnino [22] used the non-verbal audio indicators like speech, silence, pause and transition from group speech to individual speech as indicators to predict performance and expertise on a math dataset corpus of groups collaborating in solving math problems. Using these non-verbal indicators as features, they trained a model to predict the expertise of the group members and their collaborative performance. They found that these features were able to predict the expertise, but not the group performance. They did not do any analysis to find the valence of these individual audio indicators. Spikol et al. [32] used audio level and other non-verbal indicators to estimate the success of collaboration activity (i.e., measured by the human observers) while performing open-ended physical tasks around smart furniture. They found that audio level alone was sufficient to predict the quality of collaboration with high accuracy. A binary coding classification for collaboration quality was used instead of a richer set of fine-grained level of coding. Again, a deep qualitative analysis of how audio level contributed to the detection of collaboration quality was missing. Table 1 gives an overview of some of the studies on detecting the indicators of collaboration from audio and their operationalisation.

All the above studies analysed the non-verbal audio indicators (such as total speaking time, number of interruptions while speaking, overlap of speech) instead of the verbal audio indicators of collaboration. Non-verbal audio indicators of collaboration are less overt as compared to verbal audio indicators. Semantically, the content of the conversation, i.e., the verbal audio indicators of collaboration, have the same meaning most of the times.

With the rise of automatic speech recognition techniques, few studies (for example, “talk traces” [23], “meeter” [24]) took into account verbal audio indicators of collaboration. In “talk traces”, Chandrasegaran et al. [23] did topic modelling, then showed the combination of words as topic clusters and also compared it with the meeting agenda. Although topic modelling shows a representative overview of the different word clusters and their evolution during collaboration, it does not show the link between these words and the rest of the conversation, which makes it hard to understand the meeting as a whole. In “meeter” [24], the dialogues of the group members were categorized based on a collaborative task (i.e., brainstorming and sharing ideas on open-ended fixed topics in short 10 minute sessions) in a lab setting to measure information sharing and shared understanding. The number of ideas generated was an indicator of collaboration quality. They did not find any significant effects of information sharing and shared understanding on the quality of collaboration. Therefore, these controlled lab studies analysing the content of the conversation provided a high level representative overview of few topics or categories without showing the relationship between the different group members based on their conversations. Therefore, to overcome this, we describe the prototyping of our technical setup with the help of field trials in a real world setting.

## 3. The Technical Setup

In this section, we describe the technical setup: tasks undertaken, their context, architecture of the setup, data collection, pre-processing, processing and methods used for the data analysis.

### 3.1. Task Context

The collaboration task that we used as the basis for our audio recordings was to design a learning activity using the Fellowship of Learning Activity ((FOLA)^2^) (http://www.fola2.com/, last accessed on 30 April 2021) game. It is a board game [36] (e.g., an online version of the game (https://game.fola2.com/, last accessed on 30 April 2021) currently under development) played face-to-face with different themed cards and roles that is used in workshops to create awareness of the connection between learning analytics and the learning design. It also can be used as an instrument to collect indicators when planning learning analytics already while designing learning activities. This game was used in 14 face-to-face meetings (with each meeting spanning between 60 and 90 min) among different teaching staff and other staff of a university. This task had different phases, which were colour-coded based on the cards supposed to be used in that phase (as *blue, red* and *yellow*) (the phases and cards have the same meaning and are being used interchangeably henceforth) with different roles assigned to each member. The *blue* (card) phase defines the steps in the learning activity. Each learning activity consists of a sequence of interactions such as learner to teacher, learner with learning environment, material to learner and so on. The *red* phase or learning enhancing technology cards are part of the step in the game where we search for enhancements of the interactions using technology such as sensors, virtual reality, etc. The *yellow* phase defines what we want to know about the interaction or within the learning activity. For example, it can be engagement, social interaction or how students take initiative. The yellow cards can be used to get input on what teachers do in classrooms to value their design choices or actions. Each card also had some prompts to steer the group conversation.

We recorded the conversations during these meetings. The conversations were in Dutch. Each group member was pre-assigned roles during the conversation: *study coach*, *student*, *technology-enhanced learning learning analytics (TEL LA) advisor*, *game master*, *educational advisor* and *teacher*. These roles had the same meaning as a real-life student, teacher or advisor, while the game master was the main moderator of the game who also helped to steer the conversation during the task. Each group member had a clip-on microphone attached along with the respective audio recorder, which recorded and stored the conversation locally in that recorder. Next, we outline the architecture used for data collection, processing and analysis.

### 3.2. Architecture

**Figure 1:**
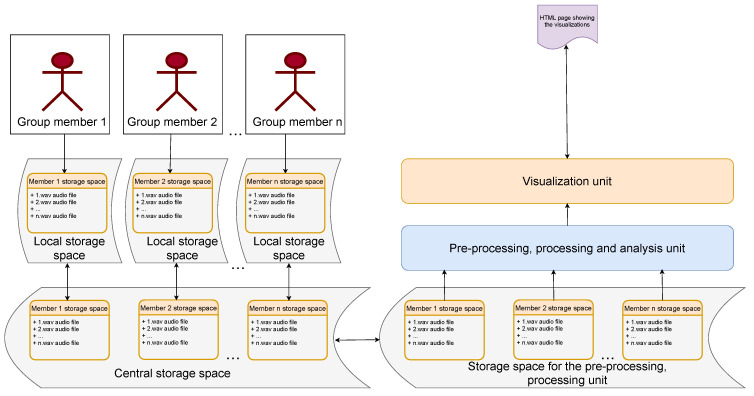
Architecture for collecting and analysing audio data during CC.

First, the audio files from each group member were saved into a storage space in the respective local device, i.e., the audio recorder. Then, after the meeting, these files were immediately transferred to the central storage space, which was the long-term storage. For the pre-processing and subsequent operations on the data, we did not disturb the original data collected, rather we took a copy of the files in the storage space for the pre-processing and processing unit. Here, we pre-processed and transcribed these audio files using Google speech-to-text. Finally, the data were processed and analysed to generate meaningful insights and passed on to the visualization unit to generate the visualizations. These visualizations were generated in a post hoc manner after the group meetings. Figure 1 shows the outline of the current architecture for collecting and analysing audio data during CC. In the subsequent sections, we describe the pre-processing, processing and analysis.

### 3.3. Data Pre-Processing and Wrangling

**Figure 2:**
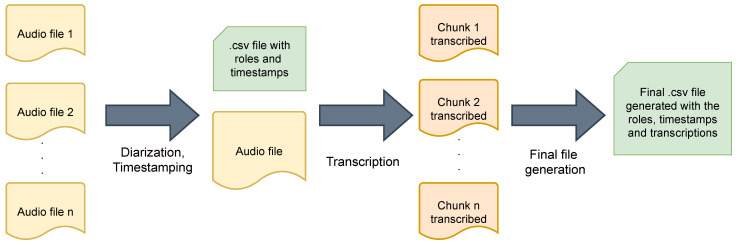
Data pre-processing schematic overview.

The data pre-processing, processing, analysis and visualizations were done in Python using different openly available libraries. We pre-processed the stored audio files for each group member by extracting the timestamp of the audio file, which denoted the exact end time of that audio file (in .wav audio file format) and the duration of the audio file. Then, we derived the start time of the audio file using the end time and the duration of the file. Next, we associated the conversations in each audio file with the group member playing a certain role, thereby associating with the group member who was speaking using *VoxSort Diarization* (https://www.voice-sort.com/, last accessed on 30 April 2021) software. This is known as speaker diarization, which helped us figure out “who spoke when?”. The group members playing specific roles were anonymized by mapping their names to roles, and these audio files were combined into one file. Finally, we transcribed this audio file using Google speech-to-text (free version) in Python. For this transcription, we split the audio file into 5 s window chunks (with a small overlap between the adjacent windows), which worked the best in our case. This helped us to increase the accuracy of the transcription, which was otherwise not good enough when we used long files of 1 to 30 min in duration. This process was repeated, and finally, these chunks were combined, sorted by timestamps from the beginning till the end of the meeting. While performing these subsequent steps, the information like timestamp and speaker role was put into the data tables in a .csv file format. Figure 2 shows the schematic overview.

**Figure 3:**
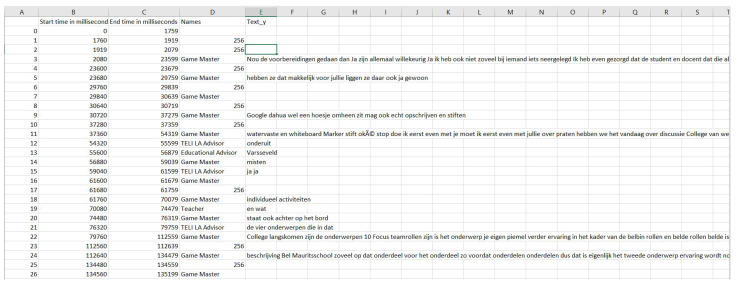
A sample from the data table in .csv format.

Then, the final extracted data along with the timestamps were stored in data tables in a .csv file format, as shown in the Figure 3. Basically, the data table had the *start time*, the *end time*, the roles of the group member under *names* (256 under names denotes noise) and the utterance as *text_y*. This data table represented the ordered conversation from the beginning till the end of the meeting. If Google speech-to-text failed to transcribe an audio file, then the corresponding text entry was left blank. Normally, this happened when a part of the audio was of a really short duration or had some random sounds like a click sound, um, claps or laughter. Now, the wrangled (cleaned, structured and enriched raw data in a desired format) data were ready for processing and analysis.

### 3.4. Data Processing

The data table stored as a .csv file was processed. Our primary focus was to analyse the content of the conversations to generate meaningful insights. To do this, we proceeded with text cleaning, processing and analysis, which come under the umbrella term of *natural language processing*. The usual approach in natural language processing is to first to clean the text. Next, we built the text model from the conversation corpus. We had to make sure that our text model could understand similarities and also understand when two different words meant similar things. Therefore, the following steps were taken by us in order to achieve this cleaning:

- Tokenization—The process of splitting sentences into good words or tokens. It lays the foundation for the next steps of cleansing.
- Elimination of stop words—The process of removing words that mean little; these are usually words that occur very frequently. Apart from using the libraries in Python for stop word removal, we also defined our list of contextual stop words that were considered unimportant for this model.
- Lemmatization and stemming—Lemmatization and stemming convert a word into its root form. For example, for the words running and runs, the stem of both words is run. Thus, after we stemmed, these words would be grouped together and retain same meaning for the model even though they had different forms.
- Sentence segmentation—We split the unstructured spoken text into different sentences, which helped the model understand the boundaries of the long text to make it more semantically distinct.
- Vectorization—Since we cannot input plain words into a model and expect it to learn from it, we had to vectorize the words. This basically means creating unit vectors for all words. As the machine can understand numbers only, so the vectorized version of words will create a dictionary for the model, which would be useful later while generating bigrams (two word combinations appearing together), trigrams (three word combinations appearing together) and topic modelling based on the keywords.

After cleaning, we proceeded with the analysis and visualizations. For the analysis, we first visualized an exploratory view of the keywords used in different phases of the CC task by different roles with the help of the cleaned text model to understand the content of the conversations. Then, we proceeded with the detailed analysis with the help of richer visualizations to understand the content and context of the conversation further with the help of our technical setup. In the subsequent sub-section, we describe the materials and methods used for the data analysis.

### 3.5. Data Analysis

**Figure 4:**
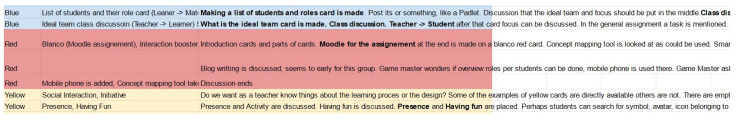
A sample annotation of the CC task.

For the scope of this article, where we describe a proof-of-concept to prototype the development of our technical setup towards automatic collaboration analytics for group speech data, we restricted our analysis to only the first out of the 14 meetings. First, we visualized in an exploratory manner to see the frequently used keywords in the text model by different group members playing different roles by using frequency analysis. To make sense of the visualizations and understand the context of the conversations, we took the help of the game master to generate summarized annotations for each phase in English (for example, a sample annotation can be seen in Figure 4).

Then, to get a representative overview of these keywords, we examined the topical clusters obtained by using LDA (latent Dirichlet allocation) and LSI (latent semantic indexing) in different phases of the meeting session. LSI helped us to identify the coherence score based on which we decided the ideal number of topics in that phase, and then, we used LDA (which is a probabilistic approach of topic modelling) in multiple iterations to find these topical clusters. LDA and LSI just show the representative keyword collection and are unsupervised algorithms for topic modelling, which can cluster semantically similar words without the need for user labelling (or input).

To go in depth into the representative overview of these words in relation to the word exchange between different roles, we looked at the different bigrams (consecutive two-word phrases) and also ranked them based on the tf-idf (term frequency-inverse document frequency) ranking. tf-idf ranking of the bigrams helped to give an overview about the frequently (with a lower tf-idf ranking) and rarely (with a higher tf-idf ranking) used bigrams. Next, we wanted to see the relationship between these words and phrases with the help of parts-of-speech tagging and construction of knowledge graphs. Knowledge graphs show the relationship between the subject, the object with the verb or the verb phrase linking them.

**Figure 5:**
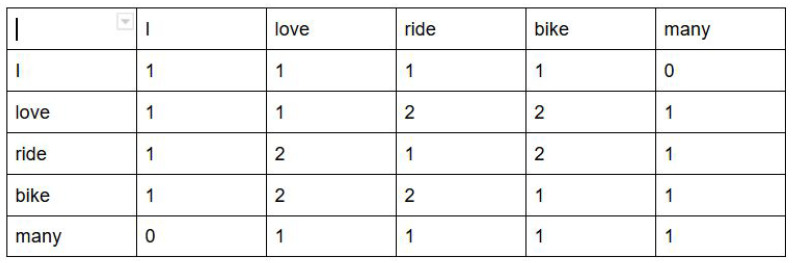
A sample co-occurrence matrix.

Knowledge graphs (as shown in the visualizations section) are sometimes difficult to interpret because of the inaccurate sentence segmentation of unstructured data. Moreover, they also do not show the strength of different words (i.e., how often these words have been used) and the strength of linkage between these words. To show this, a co-occurrence matrix was made. A co-occurrence matrix shows how many times the words co-occur in the same sentence. For example, in the two sentences: “I love riding bike” and “Bike ride is loved by many”, the co-occurrence matrix after doing text pre-processing (where we tokenized the sentences, removed stop words like “is”, “by” and lemmatized and stemmed “loved”, “riding”) would be as in Figure 5. Therefore, all the tokenized words in the text corpus are listed in rows once and again in columns, and then, the value in the co-occurrence matrix shows the number of times each word co-occurs with the other word in one sentence. Therefore, in this example, “love bike ride” is a strong combination, which is evident from the co-occurrence matrix. Machines understand this co-occurrence matrix as it shows the strength between words with the help of numbers.

Then, we visualized this co-occurrence matrix using social network analysis or the network graph. In this network graph (as shown in the visualizations section), each word from the text corpus can be shown as a node, and the edges between these nodes denote the strength between these words like how often they co-occur in the same sentence. To make the network graph visualization easier and intuitive, we built an interactive feature, which helped to highlight a specific node and its neighbours in the graph by selecting that specific node. To analyse the network graph in depth, we also looked at different centrality measures such as the betweenness centrality (BC) and eigenvector centrality (EC) of these words. Betweenness centrality shows how often a node (or word) acts as a bridge node, that is the number of times a node lies on the shortest path between other nodes. For explainability, this means that a node (or a word) with high betweenness centrality would have more control over the network. Another centrality measure that can be a good indicator of the influence of a node (or word) is eigenvector centrality. Therefore, a node with a high eigenvector centrality score must be connected to many other nodes who themselves have high scores. In the next section, we describe the visualizations generated by using these data analysis methods in the context of the first session.

## 4. Visualizations

**Figure 6:**
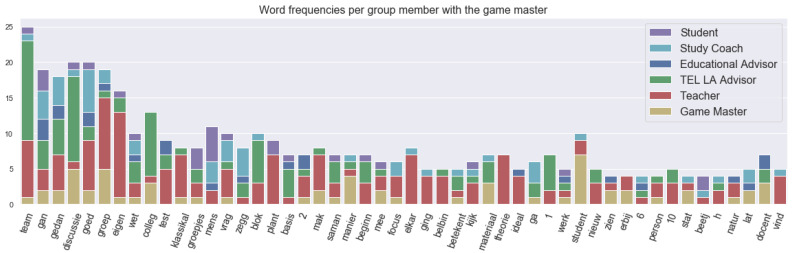
Top 50 word utterance frequency in the blue phase with roles.

First, we did an exploratory visualization using this technical setup to see the frequently used keywords in different phases by different roles. As described in the Task Context Sub-section above, in the blue phase, the main objective was to discuss the steps in the learning activity. Each learning activity consisted of a sequence of interactions such as learner to teacher, learner with learning environment, material to learner and so on. Figure 6 shows the frequency of the words used in different utterances (or spoken segments) with the roles. “Team”, “groep” and “groepjes” in Dutch mean “team”, “group” and “groups”, respectively, in English. The lemmatizer for Dutch language did not work as expected for all the words, so “groep” and “groepjes” were separate, and there were a few words like that that needed manual tweaking. The main conversation in this phase was about groups or centred on groups. The teacher and TEL LA advisor spoke mostly about groups. “Vraag” and “test” actually comprise a card “vraag test” played for the interaction between teacher and learner, which means “question test”. “Belbin” roles comprise a card for the interaction between material and learner. Belbin team roles are actually nine different team role behaviours that make a high performing team. “Blok”, “1”, “2” actually refer to the Block 1 and Block 2 cards played for the interaction between learner and learner.

**Figure 7:**
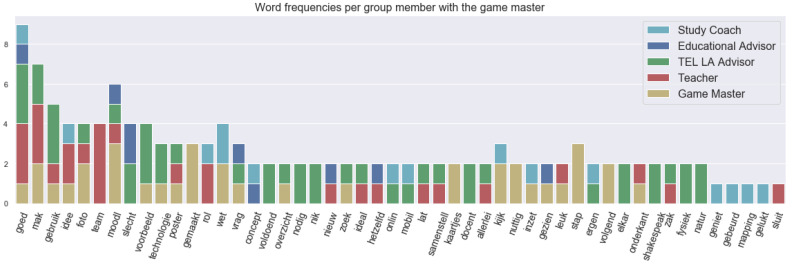
Top 50 word utterance frequency in the red phase with roles.

The red phase as defined earlier was supposed to be a conversation about learning enhancing technology. Figure 7 shows the frequency of the words used in different utterances (or spoken segments) with the roles. The Dutch term “technologie” refers to the use of technology as was desired to be found in this phase. Furthermore, TEL LA advisor fulfilled the role quite well by being the sole speaker on technology apart from the game master, who was always present in most of the discussions because of his moderating nature. Some words that related to technology or its usage were: “moodl”, “poster”, “concept”, “mapping”, “mobil” and “shakespeak”. “Moodl” refers to moodle for the assignment. The concept mapping tool was referred to by the study coach and the educational advisor, and “shakespeak” was an interaction polling system used for interactive lectures in the classroom, which can act as an interaction booster. This was referred to only by the TEL LA advisor. The use of the mobile (“mobil”) phone to take a picture of the post-its (i.e., a paper sticky note) was discussed in this phase.

**Figure 8:**
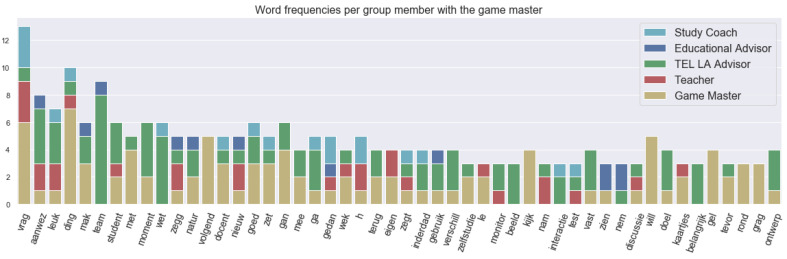
Top 50 word utterance frequency in the yellow phase with roles.

The yellow phase as defined earlier was supposed to be a conversation on the interaction within the learning activity and aspects a teacher might want to know about them. For example, it can be engagement, social interaction or how students take initiative. Figure 8 shows the frequency of the words used in different utterances (or spoken segments) with the roles. “Interactie” refers to the “interaction”, and “aanwez” means “presence”, which was at the top of the word utterance frequency because there was a specific discussion on presence and having fun, as we can see in the annotations. “Zelfstudie” and “monitor” mean “self-study” and “monitor”, respectively. They were referred to by the TEL LA advisor and the teacher during the conversation about student to material interaction.

**Figure 9:**
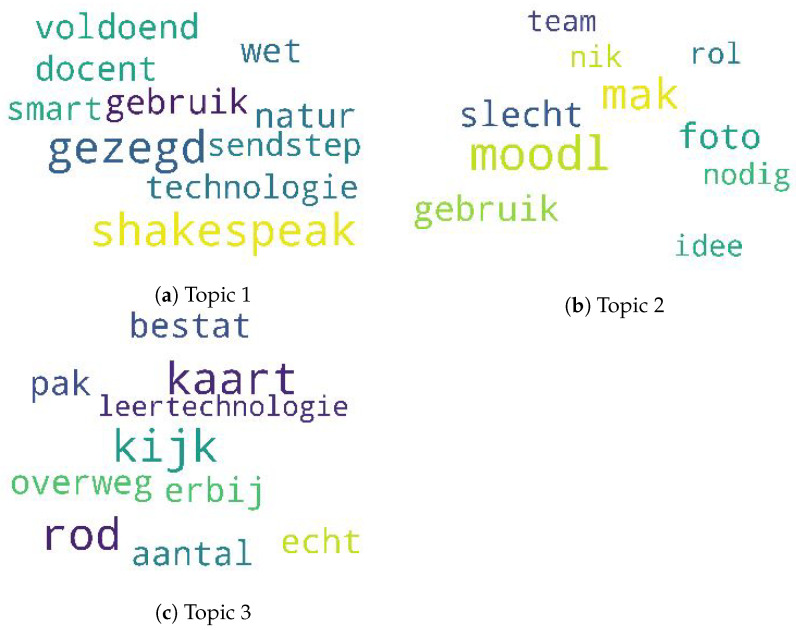
Topic clusters as word clouds in the red phase.

To get a representative overview of these keywords, we examined the topical clusters obtained by using LDA and LSI in the red phase, which had a technological underpinning. We chose to go deeper into this phase because of our inclination towards technology. Figure 9 shows the overview of the three topical cluster word clouds (with each word cloud consisting of the top 10 probable words) obtained in the red phase. As LDA and LSI just show the representative keyword collection and are unsupervised algorithms for topic modelling, we needed to label it to assign a meaning out of each cluster. Upon examining the probabilistic inclination of the topics, we found that TEL LA advisor had a higher probabilistic likelihood of getting Topic 1 as compared to other roles. Topic 1 dealt with the use of different types of interaction technology as discussed in this phase. These were mainly evident from the words: “technologie”, “shakespeak”, “sendstep” and “smart”. These technologies were to be used by the teacher while interacting with the learner, which was evident from the word “docent”, which means “teacher” in English. Therefore, some of the words in the cluster when compared with the annotations gave the meaning of the topical theme. Similarly, Topic 2 can be elaborated based on “team”, “foto”, “rol”, “moodl” and “slecht”. Topic 2 refers to the use of moodle for assignments, making a photo of the post-its using the phone. This topic cluster also captured bad (“slecht”) teams, ideas and overview roles (“rol”) per student. The last topical cluster, Topic 3, mostly focused on the use of red cards (“rod”, “kaart”) and learning technology (“leertechnologie”).

When we analysed the turn taking of different roles during the red phase, we found that the TEL LA advisor and the teacher had the most exchange of turns between them. Therefore, to further explore their roles and the usage of the words, we analysed the bigrams (two consecutive word combinations) of the words. Table 2 and Table 3 show the bigrams ranked from high to low tf-idf ranks and low to high tf-idf ranks, respectively. The tf-idf ranking tended to give a higher rank to bigrams that were used rarely and low ranks to bigrams that were used often. Therefore, from the tables, we can observe that “smart shakespeak”, “fysiek elkaar” and “goed powerpoint” were some of the top-ranked bigrams because they occurred rarely, and likewise, we also observed the low-ranked bigrams, which occurred frequently. This summarized the technology-related topics that were supposed to be discussed and also looked similar to the above topics computed by LDA. Similarly, Table 4 and Table 5 show the top-ranked and bottom-ranked bigrams respectively based on the tf-idf ranking for the teacher.

**Figure 10:**
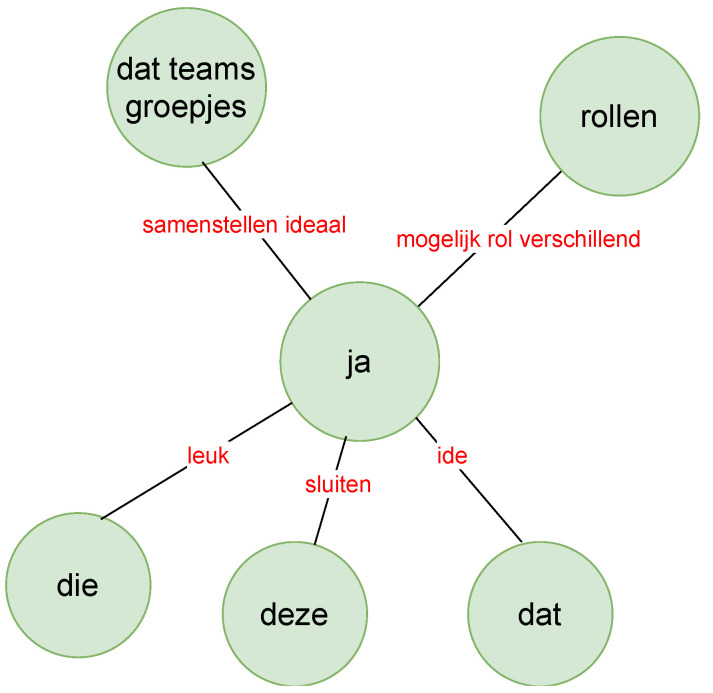
Part of the knowledge graph of the teacher in the red phase (zoomed in).

**Figure 11:**
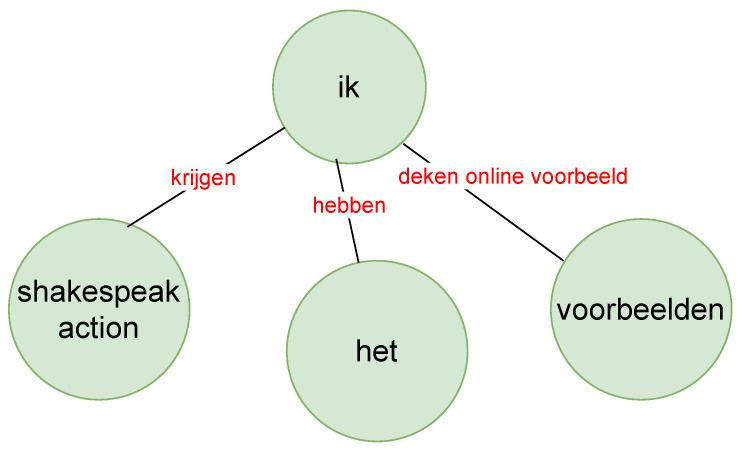
Part of the knowledge graph of the TEL LA advisor in the red phase (zoomed in).

We wanted to see the relationship between these words and phrases used by the two speakers (with the help of knowledge graphs as in Figure 10 and Figure 11) between whom most turn taking happened, i.e., the TEL LA advisor and the teacher. The green nodes show the subject and object, and the red links are the verbs or verb phrases. Although this is an interesting way to show the relationship between spoken text, it sometimes was difficult to understand the knowledge graph because of the accuracy of the sentence segmentation in the text corpus. Furthermore, we did not necessarily see the strength of the words (i.e., how often the words had been used) and the strength of the links (i.e., how often the two- or three-word phrases had been used) between these words. Therefore, we moved to the construction of a co-occurrence matrix that showed the strength of the words and also the links between words.

**Figure 12:**
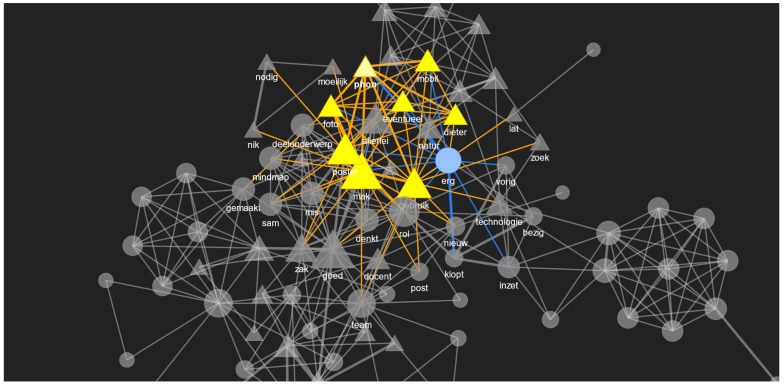
A sample social network (or network graph) of the words of the TEL LA advisor (shown as rectangles in yellow when highlighted) along with the whole red phase conversation (all other roles are shown as circles in blue when highlighted).

When there is a huge text corpus, then visualizing these relationships from a co-occurrence matrix is easier when it is displayed as a social network by using graphs with nodes and edges (as in Figure 12), where each node shows the word and its frequency reflected by the node size and the link between the nodes, i.e., the edges show the strength of the words co-occurring in the same sentence as the edge thickness. This visualization is interactive where we can select a node in the graph and highlight that node along with its neighbours. This will be helpful to get an overview of the richness of the conversations and the interaction patterns of different roles.

**Figure 13:**
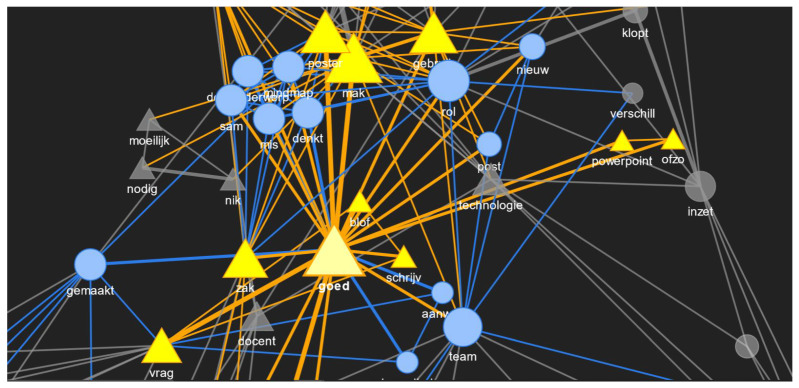
A part of the network graph with the highest betweenness centrality node (“goed”) highlighted along with its neighbouring nodes.

Moving a step further, we show a portion of the graph where the node size is proportional to the betweenness centrality, which is a better measure than the frequency of the word. Betweenness centrality shows how often a node (or word) acts as a bridge node (or a node that has more control over the network). Another centrality measure that can be a good indicator of the influence of a node (or word) is eigenvector centrality. Therefore, a node with a high eigenvector centrality score must be connected to many other nodes who themselves have high scores. Table 6 shows an overview of the comparison of the word frequency in each utterance and different centrality measures for the red phase. Figure 13 shows the connection between some words in the network graph where the node size is proportional to betweenness centrality value of that node. “Goed” had the highest betweenness centrality, and upon highlighting it, it can be seen that it is connected to “team”, which has the second highest betweenness centrality. The connection value between them is one, and the connection value between “goed” and “poster” is two. Therefore, good and poster co-occurred more in a sentence than good and team in the red phase. Out of that, “good” and “poster” as words were used by the TEL LA advisor, and “team” was not used by the TEL LA advisor at all. From Figure 7, it is clear that “team” was used by the teacher only in the red phase and by no other roles. Therefore, the purpose of these examples was to show that these graph networks can be a useful way to visualize the word importance, strength and usage by different roles during collaboration.

If EC is seen in Table 6, then “mak” and “poster” are the two words with the highest values of EC, which means that they are influential. If we refer back to the frequently occurring bigrams in Table 3 and Table 5, then “maken poster(s)” was one of the common ones for both roles who occupied most of the conversations in the red phase as computed from the frequent exchange of turns between them. This could be one of the reason for the high EC value. “Technologie” (“technology”) is in the top five of the betweenness centrality values in Table 6 even though it was not in the top five of frequency or EC, which was not surprising. This was because this red phase was about technology, and it was certain that the keyword technology would have more control over the network of words. Therefore, viewing the words, connecting words and the strength between them from the perspective of centrality could be interesting to discover latent relationships in the spoken conversations.

Thus, in this study, we took a computational approach for prototyping our technical setup and advancing towards automatic collaboration analytics.

## 5. Discussion

First, with the help of *RQ 1: “What co-located collaboration indicators have been detected from group speech data in past studies?”*, we found that most studies are on non-verbal audio indicators (e.g., total speaking time, pitch). Most previous studies on co-located collaboration (CC) focused on spectral and temporal audio indicators [16,20]. These indicators are less obvious to understand or denote what is happening during collaboration as compared to verbal audio indicators (or content of the conversation). Sometimes, depending on the cultural background of a group member, the tone of the voice can vary. Therefore, the tone of voice can be a good or bad indicator of collaboration depending on the background. On the other hand, taking the example of “what” of the conversations, it is more overt in most of the circumstances irrespective of the background. The reason for most studies being heavily inclined to the non-verbal audio indicators of collaboration could be due to the lesser maturity of automatic speech recognition (ASR) systems, making it difficult to transcribe the conversations. Of late, with the advent of different ASR systems like Google speech-to-text, it has become much easier to convert speech to text with high accuracy. Upon expanding the literature, we found very few studies (e.g., [23,24]) on verbal audio indicators of collaboration in a CC context. These studies were lab based and mostly focused on getting a representative high-level overview of the conversations as topical word clusters instead of examining the richness of the linkages among the words in the conversation.

To address this, we answered *RQ 2: “What collaboration analytics can be employed to analyse group speech data from co-located collaboration automatically?”*. For this, we built a technical setup and conducted a field trial where we recorded sessions of a board game where people had to collaboratively design learning activities and each player was assigned a specific role to play beforehand. With the help of this collaborative task, first, we had an exploratory understanding of the collected audio data set. Then, we visualized the relationship between these words, apart from the representative topic modelling (which are certain representative word clusters) done in the past [23]. First, we understood the different bigram (or two consecutive) word phrases and made a distinction between some of the most occurring bigrams and least occurring bigrams. We did this in one phase of the CC task, which was more inclined towards the technology for two roles, i.e., the TEL LA advisor and teacher, because they had the most exchange of turns during that phase. We thought this could help us uncover the main bigrams to understand the contribution of dominant turn-taking roles in that phase. Even though bigrams showed a representative collection of two consecutive word phrases, it was still difficult to understand how the conversations happened and what were the influential words (measured by eigenvector centrality), as well as what were the most controlling words (measured by betweenness centrality), which could be shown using different centrality measures, as also done in the past [37]. To understand this further, in addition to visualizing the strength of the bigrams and longer word phrases, we plotted the social network graph (as done earlier in online settings [38]), which was interactive and made it easy to select a particular node and highlight its neighbours. This network graph made it easier to understand the contribution of individual roles in the group conversation. The strength of the link between words (measured by how often they co-occurred in a sentence) along with their use by different roles helped to capture both word-level and role-level interaction, which can be a simplification approach to understand a huge text corpus. The highlighting, interactive feature of the tool was also purposefully built to reduce the information overload and only focus on that particular selected node (or word) and the neighbours automatically highlighted. Besides the visualization and its design choices, which were fully automated, during pre-processing, some degree of human involvement was necessary for sanity checks, which is explained in detail in the next section (under Challenges and Limitation).

Now, the next obvious question is: *“What is the way forward once we have this technical setup ready and a tool ready to be used in different settings?”* The first step for us is to use this across the other sessions for which we collected audio data and see if we can find some recurring themes. We also want to add further enhancements (as additional modules) and refine the visualizations by involving the two main stakeholders (i.e., group members and the person managing the CC task) for whom this was made. We want to understand the turn-taking patterns between roles further and how the conversation evolves around these turn-taking patterns. For now, it is a post hoc group collaboration analytics tool. To take it a step further, it would be interesting to add a module to detect the quality of collaboration. One possible step can be to compute the cosine similarity distance by comparing the text vectors of each role as computed by this technical setup to the expected contribution of the roles. This can give an estimate of how aligned the conversations are to the expected contribution for that role and an estimate of the collaboration quality if we can quantify the cosine similarity distance as a quality measure. Therefore, to proceed in this direction, we need to bootstrap the results of all these sessions to build models of different roles along with a human to form some expected standards (or words they would use) for each role during the CC task.

Therefore, for this article, our main contribution was twofold: identifying the gaps of the current co-located collaboration (CC) quality detection approaches from audio data and making a starting step towards an automatic holistic collaboration quality detection technical setup with the prototyping of our setup in the context of a CC task. This was a technical article stressing the different technological approaches (the coding details of which can be found on GitHub (https://bit.ly/autocollabanalytics, last accessed on 30 April 2021)) to move towards automatic collaboration analytics right from audio data collection to generating meaningful visualizations.

## 6. Challenges and Limitations

There are many challenges. First, architectural challenges are full automation, the accuracy of speaker diarization and the accuracy of speech to text. During speaker diarization, sometimes, labels of roles were misplaced, which were manually corrected. Next, there are challenges in processing and analysing the data, which are largely dependent on the accuracy of the speech to text, which we will explain below. The unstructured text data obtained from audio are much different than the data obtained from any online forums. Therefore, unstructured text data generates much noise, which to some extent can be structured by sentence segmentation. However, sentence segmentation working on only spoken text without punctuation marks or delimiters can cause sentence boundary detection problems. Another challenge in text processing is to correct the names, which were most of the time wrongly transcribed. For example, “moodle” was wrongly transcribed to “moeder”, and we had to manually fix this in the corpus. Therefore, when studies are in-the-wild without a controlled lab environment, then there are more chances for natural, unstructured conversations, which will need cleaning and structuring before analysis can yield meaningful results. The stop word corpus available to the algorithm did not remove all the contextual stop words that were not relevant for this discussion. We also needed to manually remove some contextual stop words like some action verbs depending on their importance in our context. When we lemmatized and stemmed the words, then the lemmatizer for Dutch text was not accurate enough because of its lesser usage and popularity compared to English. Therefore, we needed to manually correct some words, which could be seen in blue phase when “groep” and “groepjes” were not reduced to the same lemma as “groep”. The annotation process was time consuming.

The limitations in terms of automation can be summarized from the challenges. We needed the help of a human to pre-process to some extent for cleaning the corpus, the sanity check on the names transcribed and to make sense of the visualizations with the help of annotations. Although we are advancing towards automatic collaboration analytics, we need to eliminate other bottlenecks, especially to reduce the dependence on humans to as little as possible.

## 7. Conclusions and Future Work

First, we listed the indicators of collaboration obtained from the audio modality in the literature. We found two broad categories: non-verbal audio indicators (such as temporal, spectral audio features, total speaking time, overlap of speech) and verbal audio indicators (such as the content of the conversation, i.e., the spoken words). There have been many studies on the first category, but very few studies on the second category of analysing the content of the conversation. We found that with the maturity of automatic speech recognition systems, recently, analysis of the content of the conversations has picked up pace. Most studies analysing the content of the conversation looked at the high level topics and were lab based.

Therefore, we took a step further to build a technical setup and conducted a field trial to analyse the richness of natural unstructured conversations and the strength of the links between these words and phrases used in the conversation context, and thus, we prototyped the tool to move towards automatic collaboration analytics. Here, we analysed the conversations during a board game while designing a learning activity where group members with different roles (such as student, teacher, TEL LA advisor, study coach) interacted with each other. We found different interaction patterns between the teacher and the TEL LA advisor by analysing the word-level and phrase-level interaction during the technology related discussion phase of one collaboration session. Even though we were moving towards automated collaboration analytics, we found limitations in terms of automation with speaker diarization and data pre-processing.

As mentioned in the Discussion, we want to enhance the technical setup further by understanding the conversations around these turn-taking patterns between different roles. The major outlook for the future will be to measure the quality of collaboration and give feedback. Due to COVID-19, we are also looking into adapting our approach to a remote (or online) setting from a face-to-face setting. Because of our modular approach, it will be easier to adapt everything in the technical setup except the speaker diarization. We will not need speaker diarization in an online setting, and it will be much easier to get different clean audio streams from each group member in an online setting.

## Figures and Tables

**Table 1:** Indicators of collaboration and its operationalisation.

Parameters	Indicators	Operationalising Collaboration Quality	References
Dominance	Total speaking time	If all group members speak for almost equal total time, then there is less dominance in the group and better quality of collaboration	[5,17,18]
Active participation	Frequency of turn taking	More frequent turn changes indicate higher active participation and better quality of collaboration	[21]
Roles (one leader and other non-leaders)	Keywords used, topics covered	Closeness of the topics generated in real-time to the topics on the meeting agenda	[23]
Rapport	Synchrony in the rise and fall of the average pitch	Higher synchrony in the rise and fall of the average pitch indicates higher rapport and better collaboration quality	[20]
Expertise	Overlapped speech	Overlap in speech is an indicator of constructive problem solving, expertise and good CC quality	[25,35]

**Table 2:** Bigrams of the TEL LA advisor with high tf-idf ranking (i.e., bigrams rarely used).

Phrases (Original in Dutch)	Translated into English
smart shakespeak	smart shakespeak
fysiek elkaar	physically each other
goed powerpoint	good powerpoint

**Table 3:** Bigrams of the TEL LA advisor with low tf-idf ranking (i.e., bigrams frequently used).

Phrases	Translated into English
mobile phone	mobile phone
poster dieter	poster dieter
phone gebruiken	phone use
maken poster	make poster
gebruiken foto	use photo

**Table 4:** Bigrams of the teacher with high tf-idf ranking (i.e., bigrams rarely used).

Phrases	Translated into English
zekering interaction	certain interaction
foto maken	make photo
blok boos	block angry

**Table 5:** Bigrams of the teacher with low tf-idf ranking (i.e., bigrams frequently used).

Phrases	Translated into English
mindmap maken	make mindmap
maken posters	make posters
posters rol	posters role
samen denkt	think together

**Table 6:** Top 5 words with frequency-wise ordering, betweenness centrality (BC)-wise ordering and eigenvector centrality (EC)-wise ordering in the red phase in decreasing order. The English translation of the Dutch processed words is in the brackets.

Frequency	BC	EC
goed (good)	goed (good)	mak (make)
mak (make)	team (team)	poster (poster)
moodl (moodle)	gebruik (use)	goed (good)
gebruik (use)	technologie (technology)	rol (role)
idee (idea)	rol (role)	allerlei (all kinds of)

## Data Availability

The repositories of the algorithms, features, abstract analysis used for this study can be found in this article with link provided in the discussion section. However, we are unable to release the original audio recordings because of lack of anonymity and privacy.

## Abbreviations

The following abbreviations are used in this manuscript:

CC	Co-located collaboration
BC	Betweenness centrality
EC	Eigenvector centrality
